# Insights into the Tunnel Mechanism of Cholesteryl Ester Transfer Protein through All-atom Molecular Dynamics Simulations*[Fn FN2]

**DOI:** 10.1074/jbc.M116.715565

**Published:** 2016-05-03

**Authors:** Dongsheng Lei, Matthew Rames, Xing Zhang, Lei Zhang, Shengli Zhang, Gang Ren

**Affiliations:** From ‡ The Molecular Foundry, Lawrence Berkeley National Laboratory, Berkeley, California 94720 and; the § Department of Applied Physics, Xi'an Jiaotong University, Xi'an, Shaanxi 710049, China

**Keywords:** cholesterol, cholesterol metabolism, cholesterol regulation, cholesterol-binding protein, lipid metabolism, lipid transport, lipid-protein interaction, lipoprotein metabolism, molecular dynamics

## Abstract

Cholesteryl ester transfer protein (CETP) mediates cholesteryl ester (CE) transfer from the atheroprotective high density lipoprotein (HDL) cholesterol to the atherogenic low density lipoprotein cholesterol. In the past decade, this property has driven the development of CETP inhibitors, which have been evaluated in large scale clinical trials for treating cardiovascular diseases. Despite the pharmacological interest, little is known about the fundamental mechanism of CETP in CE transfer. Recent electron microscopy (EM) experiments have suggested a tunnel mechanism, and molecular dynamics simulations have shown that the flexible N-terminal distal end of CETP penetrates into the HDL surface and takes up a CE molecule through an open pore. However, it is not known whether a CE molecule can completely transfer through an entire CETP molecule. Here, we used all-atom molecular dynamics simulations to evaluate this possibility. The results showed that a hydrophobic tunnel inside CETP is sufficient to allow a CE molecule to completely transfer through the entire CETP within a predicted transfer time and at a rate comparable with those obtained through physiological measurements. Analyses of the detailed interactions revealed several residues that might be critical for CETP function, which may provide important clues for the effective development of CETP inhibitors and treatment of cardiovascular diseases.

## Introduction

Cardiovascular diseases cause nearly one million deaths annually in the United States (1). Two major risk factors of cardiovascular diseases are decreased levels of high density lipoprotein cholesterol (HDL-C)^3^ and elevated levels of low density lipoprotein (LDL) cholesterol (2, 3). In plasma, cholesteryl ester transfer proteins (CETPs) transfer cholesteryl esters (CEs) from high density lipoprotein (HDL) to low density lipoprotein and very low density lipoprotein (VLDL) (4). Human plasma CETP is a 476-amino acid hydrophobic glycoprotein. Genetic deficiency of CETP increases HDL-C levels and decreases LDL cholesterol levels (5, 6). As a result, CETP has become a promising drug target for the prevention and treatment of cardiovascular diseases. To date, four synthetic CETP inhibitors, torcetrapib, dalcetrapib, anacetrapib, and evacetrapib, have been evaluated in Phase III clinical studies in the past decade (7–10). The clinical data have shown that all CETP inhibitors elevate HDL-C levels, and nearly all CETP inhibitors decrease LDL cholesterol levels (7–10). Although anacetrapib is still being tested in clinical studies, the failures of torcetrapib (due to off-target effects) (7) and dalcetrapib and evacetrapib (due to their unexpected low efficacy) (8, 11) highlight that the detailed mechanism underlying the CETP-mediated transfer of CE molecules between HDL and LDL/VLDL needs to be elucidated.

Three hypotheses of the CETP mechanism have been proposed: a shuttle mechanism in which CETP collects CEs from HDL and then delivers them through the aqueous environment to LDL/VLDL (12); a ternary complex mechanism in which CETP simultaneously interacts with HDL and LDL/VLDL, thus forming a ternary complex that induces CE transfer from HDL to LDL/VLDL (13); and a modified ternary complex mechanism that implicates a CETP dimer instead of a monomer (14).

The crystal structure of CETP revealed a banana-shaped molecule composed of N- and C-terminal β-barrel domains, a central β-sheet, and an ∼60-Å-long hydrophobic central cavity (15). Two pores near the central β-sheet domain occupied by two phospholipid molecules have been suspected to be the gates between which the central cavity would interact with the aqueous environment or lipoproteins, thus favoring the shuttle mechanism (15).

Experimental studies on the kinetics of the plasma protein-catalyzed exchange of phosphatidylcholine and CE between plasma LDL and HDL have suggested that the exchange occurs by a sequential mechanism involving an HDL-CETP-LDL ternary complex (13). Recent electron microscopy (EM) studies have shown the presence of a ternary complex among CETP, HDL, and LDL/VLDL (16) in which the CETP N-terminal distal end interacts with HDL via a hydrophobic interaction (17). Independently, molecular dynamics (MD) simulations have revealed that the CETP distal ends are structurally flexible (18), and the N-terminal end penetrates into the HDL surface and takes up a CE molecule (19). A tunnel mechanism has been proposed because the CETP crystal structure displays a long hydrophobic central cavity containing two CE molecules (16). Here, we used all-atom MD simulations to evaluate the tunnel mechanism by testing whether a long tunnel could be formed and is large enough to allow CE to pass through the entire CETP molecule.

## Experimental Procedures

### 

#### 

##### Strategy for Setting Up the Simulation System

**FIGURE 1.**
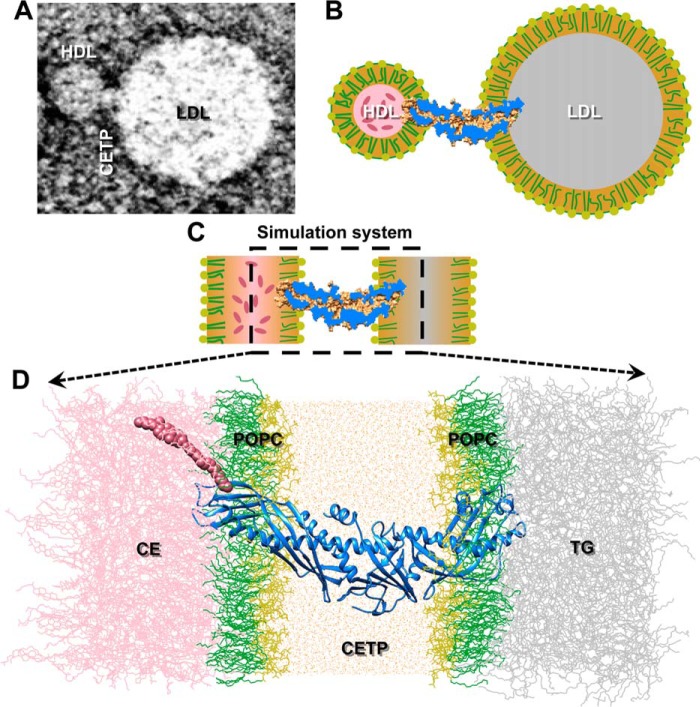
**Simulation system for studying CE transfer.** *A*, a representative negative staining EM image shows that CETP bridged HDL and LDL, forming a ternary complex (shown in schematic in *B*). *C*, a simplified simulation system (shown in schematic) was used to simulate the ternary complex to elucidate CE transfer from HDL to LDL at an atomic level. *D*, the N-terminal β-barrel domain of the CETP was inserted ∼35 Å deep into a POPC monolayer adhered to a CE pool, whereas the C-terminal β-barrel domain penetrated ∼32 Å inside an opposing POPC monolayer attached to a TG pool. The region between the two opposing lipid monolayers was filled with water molecules. The POPC headgroups and fatty tails are colored *yellow* and *green*, respectively, and the CE, TG, CETP, and water molecules are colored *pink*, *gray*, *blue*, and *orange*, respectively. The CE molecule is highlighted using van der Waals spheres.

In the tunnel mechanism, CETP interacts with HDL and LDL simultaneously and forms a ternary complex (see Fig. 1, *A* and *B*). EM experiments have suggested that the flexible N-terminal β-barrel domain of CETP penetrates into the phospholipid monolayer on the HDL surface via hydrophobic interactions (16, 17) and that the distal end reaches the HDL CE-rich pool. However, the flexible C-terminal β-barrel domain penetrates into the phospholipid monolayer of the LDL surface and interacts with the inner LDL triglyceride (TG)-rich pool (16). To test whether the ternary complex could form a tunnel within CETP that allows a CE molecule to transfer through the entire CETP molecule, we constructed an MD simulation system to match the observed EM ternary complex as closely as possible. In this system, a CETP molecule was embedded into an aqueous environment with its two distal β-barrel domain ends penetrating into the two opposite phospholipid monolayers that envelop the CE and TG pools (Fig. 1, *C* and *D*).

##### Simulation System Components

To reach equilibrium for the above simulated system within a practical period of time, our strategy was to separately equilibrate each component, *i.e.* the CE and TG pools, CETP, the phospholipid monolayers, and their simple combinations. In brief, the CE and TG pools were constructed from a non-CHARMM force field equilibration (20, 21) with a periodically expanded cross-sectional area of 96 × 96 Å. The CE pool and TG pool were then submitted for equilibration for 74.0 and 9.5 ns under a CHARMM force field (22–25) and physiological conditions using all-atom MD simulations. The equilibrated phospholipid monolayers were obtained from an equilibrated 1-palmitoyl-2-oleoyl-*sn*-glycero-3-phosphocholine (POPC) bilayer (26) that was further equilibrated for 20.0 ns by using a CHARMM force field (24, 25). All equilibrations were conducted with the Nanoscale Molecular Dynamics version 2 (NAMD2) software package (from the University of Illinois at Champaign-Urbana) (27) at 310 K and 1 atm. During the equilibrations, the 310 K temperature was maintained through Langevin dynamics (28) with a damping coefficient of 5 ps, whereas the 1-atm pressure was maintained by using the Langevin piston Nose-Hoover method (29) with a piston period of 100 fs and a decay time of 50 fs. Periodic boundary conditions and a cutoff distance of 12 Å for van der Waals interactions were applied, and the particle mesh Ewald method (30) (grid spacing <1 Å) was used to compute the long range electrostatic interactions.

The equilibrated CETP was obtained by equilibrating the CETP crystal structure (Protein Data Bank code 2OBD) (15) embedded into a cubic box containing 66,020 TIP3P (Transferable Intermolecular Potential 3 Point) water molecules and 15 Na^+^ atoms (for neutralizing the CETP surface charges). The water box boundary was at least 25 Å away from the CETP surface. The missing hydrogen atoms within the CETP crystal structure were recovered by using the AutoPSF module of the Visual Molecular Dynamics (VMD) software package (from the University of Illinois at Champaign-Urbana) (31). The system, containing a total of 206,041 atoms, was subjected to energy minimization via a total of 20,000 steps to remove the atomic clashes. Within the first 10,000 steps, the protein backbone atoms were fixed. In the next 10,000 steps, the protein backbone atoms were constrained under a force constant of 5 kcal/mol/Å^2^. The energy-minimized system was subsequently heated from 0 to 310 K over 31 ps to initiate the all-atom MD simulations. The system at 310 K and 1 atm reached equilibration after 5.4 ns; the constraints on the protein were removed after the first 0.4-ns simulation.

##### Assembling the Simulation System

The assembly process was conducted through the following three steps: (i) sandwiching the above equilibrated CE pool between two equilibrated POPC monolayers before further equilibration, (ii) sandwiching the above equilibrated TG pool with two monolayers, and (iii) allowing the two CETP β-barrel domain distal ends to penetrate into each of the above phospholipid sandwiches. The depths of penetration were ∼3–4 nm according to transmission EM measurements (16) in which the molecules clashing with CETP were removed. The simulation system, consisting of a total of ∼330,000 atoms, was then obtained after filling the gap between the two phospholipid sandwiches with equilibrated water (with 15 Na^+^ atoms for neutralization).

##### Equilibration of the Simulation System

The equilibration of the above system was achieved via three steps. (i) The overlapping molecules were manually shifted away from CETP, and then the system was subsequently subjected to 10,000 steps of energy minimization and a 0.2-ns MD simulation. This 0.2-ns simulation included a step of heating from 0 to 310 K within 62 ps and a simulation step at 310 K and 1 atm and a constraint of the heavy atoms (non-hydrogen atoms) of both water and CETP. (ii) The constraints on the water heavy atoms (oxygen) were gradually released via 10,000 steps of energy minimization followed by a 0.2-ns MD simulation. Within this step, an intermediate stage of constraint was used in which the water oxygen atoms were allowed to transfer only in parallel to the lipid monolayers under an initial force constant of 10 kcal/mol/Å^2^. (iii) The constraints on the CETP heavy atoms were gradually released via 15,000 steps of energy minimization and a 0.4-ns MD simulation. In this step, three intermediate stages were used to prevent deformations in the protein structure, *i.e.* releasing the side chain constraints, releasing non-Cα atom constraints, and gradually releasing the constraints on the Cα atoms (10 kcal/mol/Å^2^). After releasing all constraints, the system was subjected to 40-ns MD simulations at 310 K and 1 atm with NAMD2 software to achieve equilibration (27). The MD simulation used to achieve this equilibrated system was repeated three times.

##### Equilibration Analysis of the Simulation System

The equilibration analyses were conducted using VMD (31) by monitoring the changes in the following parameters. (i) The root mean square deviation (RMSD) was calculated by the spatial changes in CETP Cα atoms relative to their initial positions. (ii) The size of CETP was computed from the radius of gyration of the Cα atoms. (iii) The molecular volume was measured by using a grid size of 0.25 Å. (iv) The volumes of the CE and TG pools were estimated on the basis of the distance between the attached POPC monolayers. (v) The average distance among POPC molecules was determined from the first peak of the phosphorus radial distribution function.

The system was suggested to be equilibrated after 20 ns on the basis of the convergence analyses of the CETP and lipid structures. The CETP convergence was indicated by the RMSD (a plateau of ∼2 Å), the radius of gyration of the CETP Cα atoms, and the CETP molecular volume. The CETP structure with the lowest Cα RMSD (compared with the averaged structure within the last 20 ns of the simulation) was used to analyze the internal cavities and pore positions, which were calculated with the fpocket program with a minimum α sphere radius of 3 Å (32). The small cavities and pores containing fewer than 15 α spheres were discarded. For comparison, the cavities in the crystal structure were also identified through the same procedure. Lipid convergence was suggested on the basis of the volumes of the CE and TG pools and the average distances of the POPC molecules in each monolayer. No systematic drift was observed during the last 20 ns of the simulation.

##### Determination of the CE Transfer Pathway

Given that the CE transfer from HDL to LDL is on an approximately second time scale, a driving force was used to detect the transfer pathway within a practical time period. A CE located near but not directly contacting the distal end of the N-terminal β-barrel domain (the minimum distance between CE and CETP was more than 2.4 Å, the diameter of the hydrogen van der Waals surface) was selected as a representative molecule to probe the CE transfer pathway within the equilibrated CETP after the CEs and phospholipids it contained were removed. A step-by-step method for pathway searching was conducted as described below. When the selected CE molecule was pulled toward the center of a “pore” located in the distal end of the CETP N-terminal β-barrel domain under an example force of 8 kcal/mol/Å (applied onto the CE acyl chain end), the pore became larger (∼7 Å in diameter) and deeper (calculated by the MOLE 2.0 program (33)). The center of the deeper pore was then used as a new target to guide the driving force required to pull CE even further into CETP. After sequentially repeating the above procedures, the CE molecule was able to completely transfer through the entire CETP and exit (where the minimum distance between CE and CETP was more than 2.4 Å) from the C-terminal distal end. This process showed that CE can be transferred through a CETP molecule.

##### Transfer Time under Different Driving Forces

To test how different driving forces influence the CE transfer process, 18 different forces (6, 7, 8, 9, 10, 11, 12, 13, 14, 15, 16, 17, 18, 19, 20, 21, 22, and 23 kcal/mol/Å) were applied to the same CE molecule. Under each driving force, the MD simulations were repeated four times. Therefore, a total of 72 simulations were performed in this test. These simulations yielded a total of 72 different transfer times. The relationship between the driving force and transfer time was analyzed with MATLAB.

##### Estimation of the CE Transfer Time through Radiolabeling Experiments

To the best of our knowledge, there are no experimental data on the CE transfer time. However, the CE transfer time can be estimated from the experimental data of the CE transfer rates. Radioactive experiments have shown that the CE transfer rate with CETP is ∼75.7 ± 1.5 nmol/h/μg (34) and that the CE transfer rate with plasma is ∼64 ± 5 μg/h/ml (35), which corresponds to ∼1.14–1.54 CE molecules/s/CETP. This calculation is based on a plasma CETP concentration of ∼1.75 μg/ml (34), a CETP molecular mass of ∼73 kDa, and a CE molecular mass of ∼651 Da. Thus, the transfer time is ∼0.65–0.88 s/CE molecule/CETP.

##### Analyses of the Hydrophobicity and Diameter of the CE Transfer Pathway

The hydrophobicity of the CE transfer pathway was calculated by using a weighted averaging method and the hydrophilicities of the contact residues. The contact residues were defined as those CETP residues within a distance of less than 2.4 Å from the transferred CE at each particular pathway position. The weight of a contact residue was calculated as the ratio of the solvent-accessible surface area of the residue to the total solvent-accessible surface area of all contact residues. The hydrophilicity of a contacting residue was defined by the Kyte and Doolittle (36) scale. The diameter of the CE transfer pathway was measured with MOLE 2.0 (33) with a 3.0-Å probe radius and a 1.25-Å interior threshold.

##### Analyses of the Interaction Energies of CE-CETP and CE-Lipid along the CE Transfer Pathway

The interaction energy between the transferred CE and CETP at each particular point along the pathway was calculated by using the pair interaction function of NAMD2. The periodic boundary conditions, cutoff distance of 12 Å for van der Waals interactions, and particle mesh Ewald method for calculating long range electrostatic interactions were used for this calculation. The interaction energy between the transferred CE and all other lipids at each particular point along the pathway was calculated by the same method.

## Results and Discussion

### 

#### 

##### Simulation System Setup

To simulate CE transfer from HDL to LDL via CETP, a simulated system was established on the basis of the results of transmission EM experiments (16, 17). Transmission EM experiments have shown that the CETP N-terminal β-barrel domain penetrates into the HDL surface via a hydrophobic interaction (16, 17), whereas the C-terminal β-barrel domain penetrates into the LDL/VLDL surface; the depths of penetration are ∼3–4 nm (16). A representative transmission EM image in which a CETP penetrates into both HDL and LDL is shown in Fig. 1*A*. To simulate this CETP interaction with HDL and LDL (Fig. 1*B*), (i) the N-terminal β-barrel domain of an equilibrated crystal structure of CETP (15) was allowed to penetrate ∼35 Å into an equilibrated POPC monolayer adhering to an equilibrated CE pool. (ii) The C-terminal β-barrel domain was allowed to penetrate ∼32 Å into an opposite POPC monolayer adjacent to a TG pool (Fig. 1, *C* and *D*). (iii) The middle portion of CETP was surrounded by equilibrated and neutralized water molecules (Fig. 1*D*). This system contains a total of ∼330,000 atoms.

##### Conformational Changes of CETP

**FIGURE 2.**
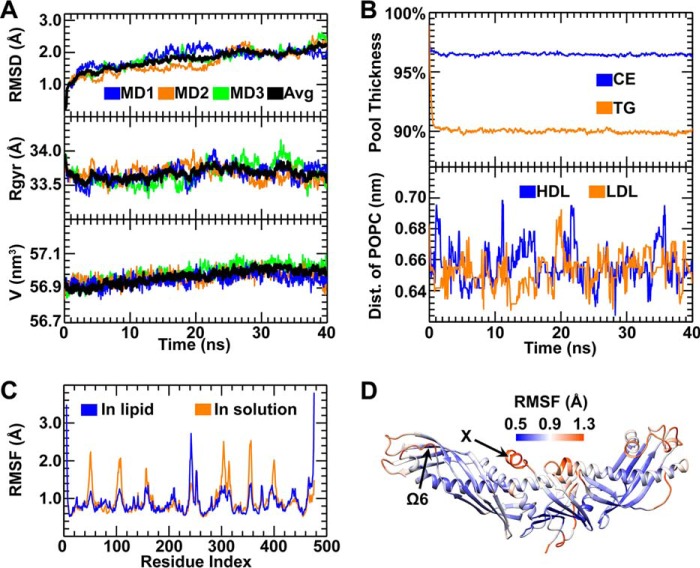
**Equilibration of the simulation system through all-atom MD simulations.** *A*, three repeated equilibrations of CETP were monitored on the basis of the RMSD (*top panel*), the radius of gyration (*Rgyr*) against the mass center of the molecule (*middle panel*), and the volume of the entire molecule (*bottom panel*). *B*, the equilibration of the CE and TG pools was achieved after a stable measured pool thickness was reached (*top panel*). The equilibration of the POPC monolayers was achieved after the distance (*Dist.*) between POPC molecules was stable (indicated by the first peak of the phosphorus radial distribution function (*bottom panel*)). *C*, the fluctuation of CETP in lipid monolayers (*blue line*), as indicated by the RMSF of the Cα atom, was calculated from the last 20 ns of simulations and compared with the RMSF of this molecule in solution (*orange line*). *D*, the CETP structure in the lipid monolayer is colored according to the RMSF values (the color and value relationship are shown as a *color bar*).

**FIGURE 3.**
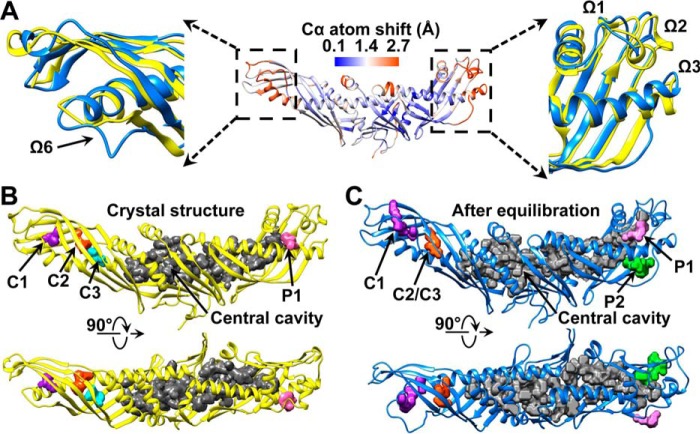
**Conformational changes in the CETP structure after equilibration.** *A*, the conformational changes in the CETP structure were determined after computing the shifts of the Cα atoms in the average structures from the last 20 ns of simulations against the crystal structure. The CETP structure is colored according to the value of the shift (the color and value relationship are shown as a *color bar*). Zoomed-in views of two predominant conformational changes located in the distal ends of the N- and C-terminal β-barrel domains are shown. These regions (*blue*) were magnified and compared with the crystal structure (*yellow*). Two perpendicular views show the CETP internal cavities and pores in the crystal structure (*B*) and the structure after equilibration in lipid monolayers (*C*).

The above system was subjected to all-atom MD simulations for 40 ns to reach an equilibrium state under physiological conditions (Fig. 2, *A* and *B*). After the simulation was repeated three times, a root mean square fluctuation (RMSF) analysis of the Cα atoms showed that CETP presented higher stability when it was embedded in the lipid monolayers (∼4% Cα atoms showed an RMSF greater than 1.4 Å) than when it was in solution (∼11% above 1.4 Å) (18). However, the distal ends of the β-barrel domains, helix X (Glu^465^–Ser^476^), and the loop connecting the two β-barrel domains (Asp^240^–Arg^259^) remained relatively flexible compared with the crystal structure (Fig. 2, *C* and *D*). Both distal ends exhibited distinguishable conformational changes (Fig. 3*A*). In the N-terminal distal end, loop Ω6 (Gln^155^–Trp^162^) shifted by a maximum distance of ∼6.2 Å (on the basis of the Cα shift) at residues Gln^155^ and Gly^156^, which directly enlarged internal cavity C1, consequently connected it to the nearby distal end pore, and indirectly connected cavities C2 and C3 (Fig. 3, *B* and *C*). These changes are similar to results from a recent MD simulation (19) where the Ω5 and Ω6 loops separate upon penetrating into HDL and open the N-terminal distal end to take up a CE molecule. This conformational change may be a result of the hydrophobic interaction between the CETP N-terminal distal end and the hydrophobic core (17). In the C-terminal distal end, a portion of helix B′ (Ile^405^–Met^412^) and the flexible regions Ω1 (Ser^286^–Thr^322^), Ω2 (Lys^347^–Thr^362^), and Ω3 (Lys^392^–Ser^404^) bend along the β-barrel domain center away from the longitudinal axis of CETP with a maximum angle of ∼6° (Fig. 3*A*, *right panel*). This bending directly generates a pore (P2) in this distal end (Fig. 3*C*), indirectly enlarges the central cavity in the central β-sheet domain, subsequently connects the central cavity to pore P2, and ultimately forms a large tunnel linked to the TG pool (Fig. 3*C*).

##### CE Transfer Strategy

To test whether a CE molecule could transfer through an entire CETP under conditions as close as possible to the real experimental conditions, an all-atom simulation method, *i.e.* a steered molecular dynamics simulation, was used. Because the time required to transfer a CE molecule from HDL to LDL is on an approximately second time scale (described under “Experimental Procedures”), all-atom simulations on such a large simulation system for nearly a second are quite challenging for the current advanced supercomputers. Although some coarse grain and implicit model methods can speed up the simulation by simplifying a group of atoms as one united bead or the solvent/lipid as a continuous medium and are highly successful in simulating protein dynamics, modeling, design, and prediction (37, 38), this simplification may also reduce the accuracy of the simulation and distort the protein structure. For example, an implicit model for both the solvent and lipids has been shown to fail to maintain the structure of the transmembrane helix dimers of ErbB1/B2 and EphA1, potentially causing kinking, bending, or even twisting within the structure of these helices (39). Therefore, an all-atom simulation is still required to simulate the detailed process of CE transfer. However, we must find a practical solution tailored to the limitations of current computational capabilities regarding simulation time.

Our intermediate solution was to introduce a driving force on the transferred CE molecule to speed up the CE transfer process on the basis of the following reasons. (i) The transfer process involves no chemical reaction or external energy. (ii) The chemical properties of HDL and LDL are similar (40, 41). (iii) CE transfer from HDL to LDL is likely to be a kinetic process (13). We believe that the differences in the physical properties of HDL and LDL, such as the inner pressures and CE concentrations, play a key role in generating a driving force for CE transfer. Experimental measurements have shown that HDL and LDL have a similar surface tension (42–44). According to the Young-Laplace equation, the smaller the particle, such as HDL, the higher the internal pressure over a larger particle, such as LDL, under the same surface tension. This difference in internal pressure would generate a force to drive CE transfer.

To avoid a potential error induced by using a specific force, a series of driving forces were also applied to the transferred CE. By statistically analyzing the simulation results under different driving forces, we may gain a new understanding of the detailed transfer process and mechanism.

##### Probing the CE Transfer Pathway

**FIGURE 4.**
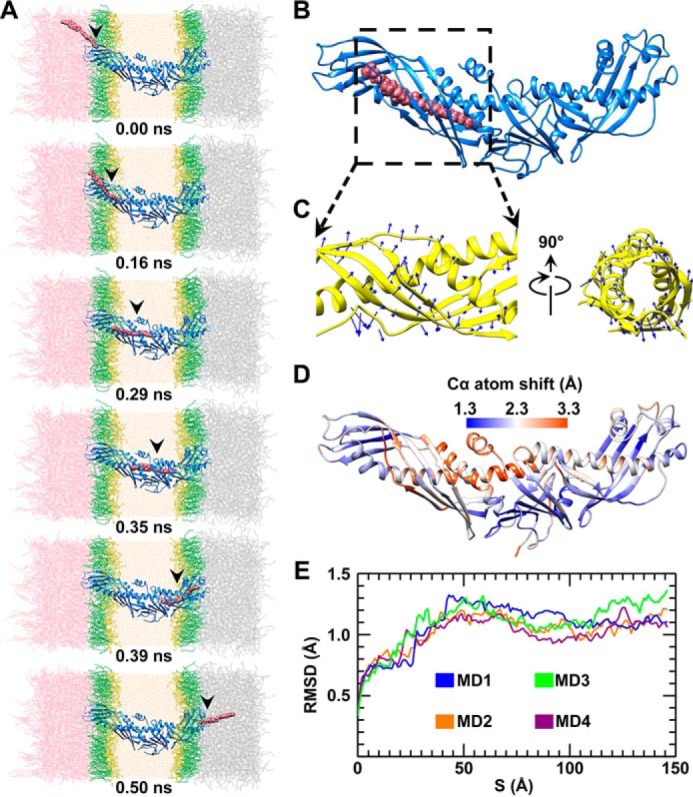
**CE transfer through the CETP molecule through all-atom steered molecular dynamics simulations.** *A*, snapshots of a CE molecule (shown in van der Waals spheres) during transfer through a CETP molecule (shown in *ribbon*) under a representative force of 11 kcal/mol/Å. The POPC headgroups and fatty tails are colored *yellow* and *green*, respectively, and the CE, TG, CETP, and water molecules are colored *pink*, *gray*, *blue*, and *orange*, respectively. Shown is a representative snapshot image of CETP when the CE molecule was in the middle of the N-terminal β-barrel domain (*B*), leading to the rotation of the N-terminal β-barrel domain (*C*, *blue arrows*). *D*, each residue shifts from its original position during CE transfer. For each Cα atom, the maximum shift from four simulations was averaged and colored on the CETP structure (the color and value relationship are shown as a *color bar*). *E*, CE transfer through CETP was simulated four times under the representative force of 11 kcal/mol/Å. The RMSD from each simulation *versus* the CE position along the CE transfer pathway is shown.

**FIGURE 5.**
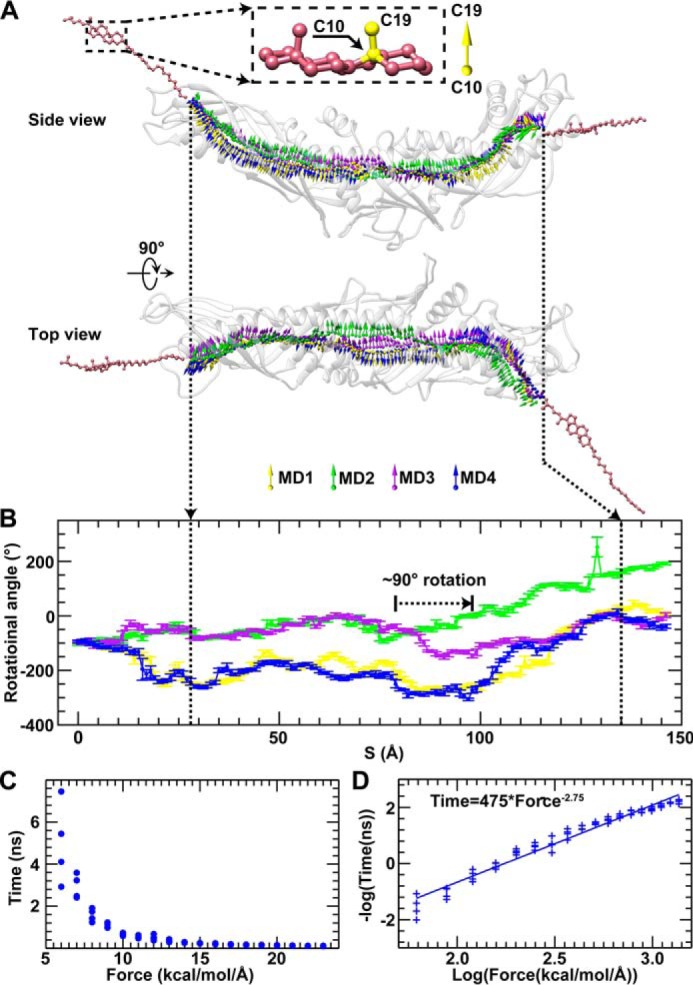
**CE transfer pathways and the relationship between transfer time and driving force.** *A*, the transfer processes described above were repeated four times. The CE transfer pathways are shown by the trajectories of the C10–C19 bond of the CE steroid ring during its transfer within the CETP tunnel. Each pathway of CE transfer is shown in different colors. The rotational angle of the C10–C19 bond against the paper surface in the side view was used as an indicator of the CE steroid ring orientation (*B*). *Error bars* represent S.D. The CE steroid ring would rotate ∼90° to pass through the central cavity and exit the C-terminal distal pore. After repeating the CE transfer 72 times under a series of 18 driving forces (from 6 to 23 kcal/mol/Å) (*C*), the relationship between CE transfer time and driving force is plotted according to the negative log and subsequently fitted using a linear equation (*D*). The best fit line has a slope of 2.75 and an *R*-factor of 0.96, corresponding to the following time and force relationship: Time (ns) = 475 × Force (kcal/mol/Å)^−2.75^.

Under a representative force of 11 kcal/mol/Å, a CE molecule was pulled to the surface pore at the CETP N-terminal distal end (prior to this simulation, two phospholipids and two CE molecules inside this CETP were removed). When CE penetrated the pore (Fig. 4*A*), a deeper pore was generated via the rearrangement of the nearby side chains of CETP. Through the continued pulling of CE deeper into the pore (Fig. 4*B*), additional conformational changes were induced in CETP (Fig. 4*C*), *i.e.* a roughly counterclockwise rotation of the N-terminal β-barrel domain, as proposed previously (16). This rotation connected the pore to the nearby internal cavities C2 and C3 and formed a connection to the central cavity within the central β-sheet domain. By sequentially pulling the CE through CETP, this molecule eventually exited from the C-terminal distal pore after a total simulation time of ∼0.5 ns (Fig. 4*A*; before and after transfer, there is no physical contact between CE and CETP). This process was repeated four times (Figs. 4*E* and 5*A*), and each time, we confirmed that a CE molecule could be transferred through the entire CETP molecule (Figs. 4*A* and 5*A* and supplemental Movie 1).

Although the CE orientations during the four repeated simulations (indicated by the direction of the C10–C19 bond shown in Fig. 5*A*) were flexible before entering the CETP and after exiting from the CETP, the CE steroid ring exhibited relatively constrained orientations that were parallel to each other during CE transfer within the N- and C-terminal β-barrel domains (Fig. 5, *A* and *B*). Notably, CE made an ∼90° turn near the central β-sheet, a result consistent with the observed ∼90° difference in orientations between the two CEs within the crystal structure (15).

##### Analysis of CE Transfer Time

To confirm the ability of the CE molecule to transfer through the entire CETP molecule, a series of different driving forces (from 6 to 23 kcal/mol/Å with increments of 1 kcal/mol/Å) were sequentially applied to the CE molecule. Under each driving force, the simulations were repeated four times. In all of the 72 simulations, the CE molecule was confirmed to transfer through the entire CETP molecule, and the average transfer times under each driving force were 4.98 ± 1.94, 2.93 ± 0.57, 1.58 ± 0.30, 1.08 ± 0.12, 0.66 ± 0.06, 0.54 ± 0.06, 0.51 ± 0.13, 0.35 ± 0.06, 0.28 ± 0.02, 0.24 ± 0.01, 0.21 ± 0.02, 0.18 ± 0.01, 0.16 ± 0.01, 0.15 ± 0.01, 0.14 ± 0.01, 0.13 ± 0.01, 0.11 ± 0.01, and 0.10 ± 0.01 ns (mean ± S.D.) from 6 to 23 kcal/mol/Å, respectively (Fig. 5*C*). Statistical analysis of the relationship between the driving force and transfer time showed that the negative log of the transfer time presented a linear relationship with the log of the driving force with an *R*-factor of 0.96 and a slope of 2.75 (Fig. 5*D*).

From the above relationship between the driving force and transfer time, we predicted that the CE transfer time should be ∼0.008–0.03 s/CE molecule (*i.e.* ∼33–125 CE molecules/s/CETP) under an estimated driving force of ∼0.018–0.029 kcal/mol/Å. The exact experimental driving force on the transferred CE has not been measured. However, we were able to estimate the driving force by using the method described below. The driving force should be calculated on the basis of the different chemical potentials between HDL and LDL. The chemical potential μ is defined as *U* + *PV* − *TS* where the *U* is the internal energy (related to the chemical reaction and phase transition); *P* and *V* are the pressure and volume, respectively; and *T* and *S* are the temperature and entropy, respectively. Given that the chemical components within HDL and LDL are similar (neutral lipid core, *i.e.* CEs and TGs, surrounded by a phospholipid monolayer and amphipathic apolipoproteins) and that there is no relevant chemical reaction, temperature change, phase transition, or net CE volume change during CE transfer, the differences in the pressures and entropies would dominate the difference in chemical potential. HDL should have a higher chemical potential than LDL because of its higher internal pressure (discussed above) and lower entropy (*i.e.* higher CE concentration).

From the internal pressure differences and the dimensions of the CETP tunnel (∼6 Å measured using the MOLE 2.0 program, which is similar to that of the steroid ring of CE, *i.e.* ∼6 Å in width and ∼4 Å in thickness (15)), we calculated that the driving force is ∼0.018–0.029 kcal/mol/Å. The inner pressure difference was calculated by using the Young-Laplace equation (*P* = 2α/*R* where α is the surface tension and *R* is the radius of the particle) and the following experimental results. (i) The surface tensions of HDL and LDL are both ∼0.020–0.033 newton/m according to previous surface balance and surface radioactivity experiments (42–44). (ii) The average diameters of HDL and LDL are ∼100 (45) and ∼220 Å (46), respectively.

On the basis of the above driving force, the transfer times were predicted to be ∼0.008–0.03 s/CE molecule (*i.e.* ∼33–125 CE molecules/s/CETP). This transfer time is shorter than the value that was calculated from the radiolabeled CE experiments with the plasma CETP concentration (34, 35), *i.e.* ∼0.65–0.88 s/CE molecule or ∼1.14–1.54 CE molecules/s/CETP molecule. Because the time spent for CETP travel, sensing, interacting, and penetrating both HDL and LDL was not included in the predicted transfer time from our simulations, a longer time should be expected for CE transfer under physiological conditions.

##### Interaction Energy between CE and CETP

Calculation of the free energy is an ideal method to analyze the system energy. This free energy calculation, particularly the portion attributed to entropy (47), is practically impossible in our simulated system containing ∼34,000 molecules with multiple dimensions of freedom (such as the internal bending and rotational degrees of freedom of the transferred CE molecule) because of the limitation of computational power. Although a fast and easy estimation of free energy can be achieved by using an implicit solvent model, the model may also distort the protein structure as described previously (39). Therefore, we chose the most reliable all-atom simulation result to calculate two specific interaction energies (*i.e.* CE-CETP and CE-lipid) as a part of our energy analyses.

**FIGURE 6.**
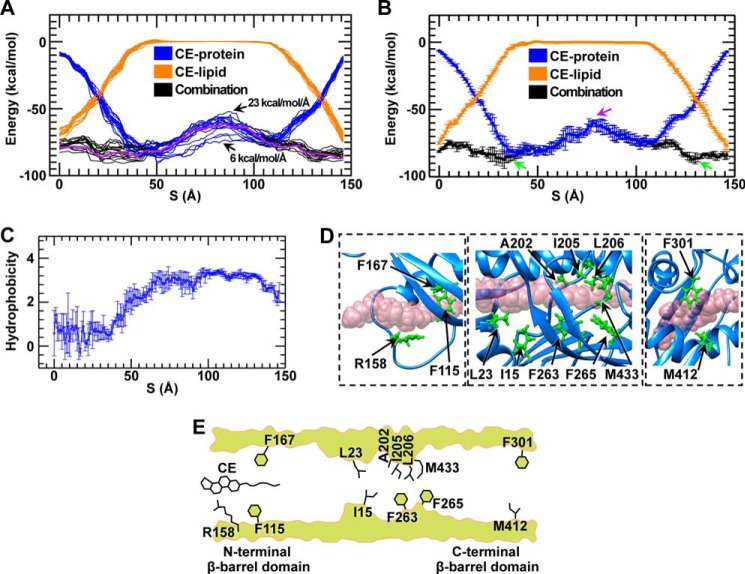
**Energy barriers, energy wells, and residues required for CE transfer through CETP.** *A*, energy distributions along the CE transfer pathway under a series of driving forces (ranging from 6 to 23 kcal/mol/Å). Each curve was averaged from four experiments repeated with the same driving force. The combination energy (*black line*) includes the interaction energies, *i.e.* the energy between the transferring CE molecule and lipid pools (*orange line*) and the energy between the transferring CE molecule and the CETP molecule (*blue line*). The energy distribution was calculated within a 6-Å step along the transfer pathway. The combined energy under a representative driving force of 11 kcal/mol/Å is highlighted in *purple*. The energy under the driving forces of 6 and 23 kcal/mol/Å is indicated by *arrows. B*, the representative energy distributions under a driving force of 11 kcal/mol/Å are shown with *error bars* indicating S.D. calculated from four repeated simulations within a 1-Å step along the transfer pathway. The central energy barrier and low energy wells within the β-barrel domains are indicated by *purple* and *green arrows*, respectively. *C*, the hydrophobicity of the tunnel wall surrounding the transferred CE molecule (distance <2.4 Å) is displayed as the average hydrophobicity within a 1-Å step against the CE transfer pathway. *Error bars* represent S.D. *D*, the residues that predominantly contributed to the energy wells in the N-terminal and C-terminal β-barrel domains (*left* and *right panels*) and to the energy barrier in the central region (*central panel*) are highlighted in *green. E*, a schematic showing the residues that might regulate CE transfer via strong interactions.

Under a representative driving force of 11 kcal/mol/Å, the all-atom simulation results were used to compute the interaction energy between the transferred CE and CETP against the CE transfer pathway (Fig. 6, *A* and *B*, *blue lines*). The energy distribution showed that CE exhibited a higher CE-CETP interaction energy, which quickly decreased when CE penetrated into the CETP tunnel. In the middle of the tunnel, this interaction energy was relatively flat except for a relatively high energy barrier that was observed near the central β-sheet where the CE steroid ring turned ∼90° to pass through this portion of the tunnel (Fig. 5, *A* and *B*).

##### Interaction Energy between CE and Lipids

Similarly, we used the same all-atom simulation result to analyze the interaction energy between the transferred CE and all other lipids (Fig. 6, *A* and *B*, *orange lines*). The energy distribution throughout the transfer pathway showed that CE exhibited a lower energy when it was close to either side of the neutral lipid pools and exhibited a higher energy when it was located between the neutral lipid pools.

The combined energy of the CE-CETP and CE-lipid showed a much more flat distribution than did either the CE-CETP or CE-lipid interaction. Notably, the combination energies were similarly low at the positions before CE penetration or after CE exit from the CETP tunnel, thus suggesting that CE transfer required nearly no initial potential energy. When CE was transferring into the tunnel, the combination energy was dominated by the CE-CETP interactions in which the highest interaction energy was around the central tunnel region. These combination energy distribution profiles were similar to one another under different driving forces. The central energy barrier was maintained at the same position even after the driving force was decreased to 6 from 23 kcal/mol/Å (Fig. 6*A*), which corresponded to a ∼50-fold increase in simulation time. The similarities in energy profiles and barrier locations reflected that our simulations may reveal intrinsic information about CE transfer through the CETP tunnel.

##### Residues Associated with the Energy Barrier and the Energy Wells

Although the combined energy distribution was relatively low and flat (Fig. 6, *A* and *B*, *black line*) compared with either the CE-lipid or CE-CETP energy, a relatively high energy barrier was observed when CE was located near the central tunnel region. To determine the residues that might contribute to this energy barrier, we conducted the following analyses based on hydrophobicity and geometrics.

The hydrophobicity analysis showed that the entire tunnel/pathway is relatively hydrophobic (Fig. 6*C*), thus providing the condition required for CE transfer through CETP. The geometric analysis showed that the tunnel diameter near the central region was only ∼6.0 ± 0.6 Å. This narrow region (called the “neck” of the tunnel in the crystal structure (15)) is similar to or slightly larger than the width of a CE molecule (∼6 Å wide and ∼4 Å thick (15)), thus suggesting that it may be possible to transfer an entire CE molecule through this region (Fig. 6*D*). We found that the residues surrounding this narrow region (within ∼10-Å distance range), including Ile^15^, Leu^23^, Ala^202^, Ile^205^, Leu^206^, Phe^263^, Phe^265^, and Met^433^ (Fig. 6*D*, *central panel*), participated in physical contacts with the CE molecule (distance within 2.4 Å, the diameter of a hydrogen van der Waals surface), which might contribute to this local high energy barrier.

In addition to the above energy barrier, a low energy well was observed within both the N-terminal and C-terminal β-barrel domains (Fig. 6*B*). The Phe^115^, Arg^158^, and Phe^167^ residues contributed the greatest energy to the N-terminal energy well (Fig. 6*D*, *left panel*), which may attract CE molecules from the CE pool into the CETP tunnel. The Phe^301^ and Met^412^ residues contributed the greatest energy to the C-terminal energy well (Fig. 6*D*, *right panel*), which may orient or facilitate the rotation of the CE steroid ring to allow exit from the C-terminal pore (Fig. 5*A*).

Because the residues identified above can form physical contacts or strong interactions with the transferred CE, mutation of these residues may change their contacts or interactions with CE, thus affecting the CE transfer rate. Consistent with this hypothesis, experiments have shown that mutating the Ile^15^ and Met^433^ at the CETP central narrow region to the bulky residue tryptophan (*i.e.* I15W and M433W) and the F301D mutation at the C-terminal distal end decreases CETP transfer activities by ∼60, ∼40, and ∼20%, respectively (15). For the other identified residues, there have been no reported results from mutational experiments to the best of our knowledge. Additional mutational analyses of these residues are needed to further verify the tunnel transfer mechanism. Notably, the mutations of several residues, such as L468Q (48), T138Y, and L457W (15), have shown effects on CE transfer. However, those residues were not identified in our energy and physical interaction analyses. The failure to identify these residues may be because only the mutated residues may form direct interactions with the transferred CE, or their mutation may generate a secondary effect on other nearby residues that interact with the transferred CE.

##### CE Transfer Mechanisms

Our previous EM studies have demonstrated the existence of a ternary complex of HDL-CETP-LDL/VLDL (16), suggesting a tunnel mechanism for CETP. The simulations indicated that it is possible for a hydrophobic CE molecule to transfer through an entire CETP via a continuous hydrophobic tunnel between the two distal ends of CETP (Fig. 6*E*). This tunnel might facilitate CE uptake into the tunnel without using energy. Upon entering the tunnel, hydrophobic interactions among the CE fatty tails and residues Phe^115^ and Phe^167^ may mediate the penetration of the CE molecule further into the tunnel. During subsequent penetration of the hydrophobic CE molecule through this hydrophobic tunnel, a narrow region might act as a rate-limiting site, leading to a prominent energy barrier for CE transfer. The internal pressure difference between HDL and LDL might provide the additional energy required for CE to pass through this energy barrier. Upon passing through this region, no further major energy barrier would prevent the exit of CE from the C-terminal opening. Residues Phe^301^ and Met^412^ may even assist the exit of CE by facilitating the rotation of the CE steroid ring. This observed CE flow from HDL to LDL requires no external energy input, thus suggesting that CE may transfer through the CETP tunnel by diffusion.

The prominent interaction energy barrier for CE transfer through CETP is ∼21 kcal/mol (Fig. 6*B*) (estimated under a driving force of 11 kcal/mol/Å and transfer time of ∼0.5 ns and without including entropy). To understand this barrier, we estimated the energy barrier for CE transfer into an aqueous phase through a lipid monolayer. It has been reported that the peak free energy barrier is ∼140 kJ/mol (*i.e.* ∼33.5 kcal/mol) for transfer of a cholesteryl oleate through a 1,2-dimyristoyl-*sn*-glycero-3-phosphocholine lipid monolayer into an aqueous phase (49, 50) in which the interaction energy (without including the entropy) has been estimated to be ∼28.6 kcal/mol. This energy barrier is higher than our energy barrier. Moreover, because the transfer time (under a driving force of 11 kcal/mol/Å) is 10^9^–10^10^ times faster than the transfer time measured experimentally (approximately seconds) (34, 35), the energy barrier under the experimental transfer time should be even much lower than ∼21 kcal/mol, thus suggesting that it would be even easier for the CE molecule to transfer through the CETP molecule than through the lipid monolayer into an aqueous phase.

Although the tunnel model was favored by our test, some other experiments have not favored this model. For example, by incubating small peptides of the CETP C terminus with HDL (49), small micelle-like structures have been generated through secondary structure disorder-to-order transitions of these small peptides. The authors of this previous study have proposed a small micelle-like structure transfer model in which those micelle-like structures mediated CE transfer from HDL to LDL/VLDL. In this proposed model, the authors have not explained how those micelle-like particles, which contain no protein, could function to sense, target, and bind to LDL/VLDL (instead of HDL, other plasma vesicles, and cells in plasma) for directional CE transfer. Moreover, these micelle-like structures have not been observed after incubation of full-length CETP with HDL (16, 17). A recent, independent EM study has concluded that CE transfer does not require a ternary tunnel complex with CETP according to negative evidence, *i.e.* the lack of an observation showing the existence of the CETP-LDL complex or the HDL-CETP-LDL complex (51). The weakness of the logic in this study is that this failure to observe the complex has been used as evidence of its non-existence instead of being used as non-supporting evidence.

Although our simulations support the possibility of CE transfer through the CETP tunnel, a study directly observing the CE molecule transfer process or more convincing experiments are still necessary to validate this mechanism. An indirect validation study could be designed by using new CETP inhibitors to target the CETP hydrophobic tunnel, particularly the narrow central region and distal end pore regions, to introduce energy barriers for CE passage. Our insights into the CE transfer pathway through CETP may provide important clues for the design of new CETP inhibitors to efficiently treat cardiovascular diseases.

## Author Contributions

G. R. initiated and designed this project, and D. L., S. Z., X. Z., M. R., and L. Z. modified this project. D. L. performed the modeling, MD simulations, and data analyses. D. L., M. R., and G. R. interpreted the data and drafted the initial manuscript, and D. L., X. Z., M. R., L. Z., G. R., and S. Z. revised the manuscript.

## Supplementary Material

Supplemental Data

## Footnotes

3 The abbreviations used are:

HDL-C: high density lipoprotein cholesterol
CE: cholesteryl ester
CETP: cholesteryl ester transfer protein
MD: molecular dynamics
POPC: 1-palmitoyl-2-oleoyl-*sn*-glycero-3-phosphocholine
RMSD: root mean square deviation
RMSF: root mean square fluctuation
TG: triglyceride.

## References

[1] Mozaffarian D., Benjamin E. J., Go A. S., Arnett D. K., Blaha M. J., Cushman M., de Ferranti S., Després J. P., Fullerton H. J., Howard V. J., Huffman M. D., Judd S. E., Kissela B. M., Lackland D. T., Lichtman J. H., et al (2015) Heart disease and stroke statistics—2015 update: a report from the American Heart Association. Circulation 131, e29–e322 25520374 10.1161/CIR.0000000000000152

[2] Gordon T., Castelli W. P., Hjortland M. C., Kannel W. B., and Dawber T. R. (1977) High density lipoprotein as a protective factor against coronary heart disease. Am. J. Med. 62, 707–714 193398 10.1016/0002-9343(77)90874-9

[3] Camejo G., Waich S., Quintero G., Berrizbeitia M. L., and Lalaguna F. (1976) The affinity of low density lipoproteins for an arterial macromolecular complex. A study in ischemic heart disease and controls. Atherosclerosis 24, 341–354 184797 10.1016/0021-9150(76)90126-x

[4] Tall A. R. (1986) Plasma lipid transfer proteins. J. Lipid Res. 27, 361–367 3522782

[5] Brown M. L., Inazu A., Hesler C. B., Agellon L. B., Mann C., Whitlock M. E., Marcel Y. L., Milne R. W., Koizumi J., Mabuchi H., Takeda R., and Tall A. R. (1989) Molecular basis of lipid transfer protein deficiency in a family with increased high-density lipoproteins. Nature 342, 448–451 2586614 10.1038/342448a0

[6] Sakai N., Matsuzawa Y., Hirano K., Yamashita S., Nozaki S., Ueyama Y., Kubo M., and Tarui S. (1991) Detection of two species of low density lipoprotein particles in cholesteryl ester transfer protein deficiency. Arterioscler. Thromb. 11, 71–79 1988006 10.1161/01.atv.11.1.71

[7] Barter P. J., Caulfield M., Eriksson M., Grundy S. M., Kastelein J. J., Komajda M., Lopez-Sendon J., Mosca L., Tardif J. C., Waters D. D., Shear C. L., Revkin J. H., Buhr K. A., Fisher M. R., Tall A. R., et al (2007) Effects of torcetrapib in patients at high risk for coronary events. New Engl. J. Med. 357, 2109–2122 17984165 10.1056/NEJMoa0706628

[8] Schwartz G. G., Olsson A. G., Abt M., Ballantyne C. M., Barter P. J., Brumm J., Chaitman B. R., Holme I. M., Kallend D., Leiter L. A., Leitersdorf E., McMurray J. J., Mundl H., Nicholls S. J., Shah P. K., et al (2012) Effects of dalcetrapib in patients with a recent acute coronary syndrome. New Engl. J. Med. 367, 2089–2099 23126252 10.1056/NEJMoa1206797

[9] Gotto A. M. Jr., and Moon J. E. (2012) Safety of inhibition of cholesteryl ester transfer protein with anacetrapib: the DEFINE study. Expert Rev. Cardiovasc. Ther. 10, 955–963 23030283 10.1586/erc.12.82

[10] Nicholls S. J., Brewer H. B., Kastelein J. J., Krueger K. A., Wang M. D., Shao M., Hu B., McErlean E., and Nissen S. E. (2011) Effects of the CETP inhibitor evacetrapib administered as monotherapy or in combination with statins on HDL and LDL cholesterol: a randomized controlled trial. JAMA 306, 2099–2109 22089718 10.1001/jama.2011.1649

[11] Jarvis L. (2015) Lilly pulls heart drug. Chem. Eng. News 93, 9

[12] Barter P. J., and Jones M. E. (1980) Kinetic studies of the transfer of esterified cholesterol between human plasma low and high density lipoproteins. J. Lipid Res. 21, 238–249 7373163

[13] Ihm J., Quinn D. M., Busch S. J., Chataing B., and Harmony J. A. (1982) Kinetics of plasma protein-catalyzed exchange of phosphatidylcholine and cholesteryl ester between plasma lipoproteins. J. Lipid Res. 23, 1328–1341 7161562

[14] Tall A. R. (1993) Plasma cholesteryl ester transfer protein. J. Lipid Res. 34, 1255–1274 8409761

[15] Qiu X., Mistry A., Ammirati M. J., Chrunyk B. A., Clark R. W., Cong Y., Culp J. S., Danley D. E., Freeman T. B., Geoghegan K. F., Griffor M. C., Hawrylik S. J., Hayward C. M., Hensley P., Hoth L. R., et al (2007) Crystal structure of cholesteryl ester transfer protein reveals a long tunnel and four bound lipid molecules. Nat. Struct. Mol. Biol. 14, 106–113 17237796 10.1038/nsmb1197

[16] Zhang L., Yan F., Zhang S., Lei D., Charles M. A., Cavigiolio G., Oda M., Krauss R. M., Weisgraber K. H., Rye K. A., Pownall H. J., Qiu X., and Ren G. (2012) Structural basis of transfer between lipoproteins by cholesteryl ester transfer protein. Nat. Chem. Biol. 8, 342–349 22344176 10.1038/nchembio.796 PMC3792710

[17] Zhang M., Charles R., Tong H., Zhang L., Patel M., Wang F., Rames M. J., Ren A., Rye K. A., Qiu X., Johns D. G., Charles M. A., and Ren G. (2015) HDL surface lipids mediate CETP binding as revealed by electron microscopy and molecular dynamics simulation. Sci. Rep. 5, 8741 25737239 10.1038/srep08741 PMC4348656

[18] Lei D., Zhang X., Jiang S., Cai Z., Rames M. J., Zhang L., Ren G., and Zhang S. (2013) Structural features of cholesteryl ester transfer protein: a molecular dynamics simulation study. Proteins 81, 415–425 23042613 10.1002/prot.24200 PMC3557553

[19] Cilpa-Karhu G., Jauhiainen M., and Riekkola M. L. (2015) Atomistic MD simulation reveals the mechanism by which CETP penetrates into HDL enabling lipid transfer from HDL to CETP. J. Lipid Res. 56, 98–108 25424006 10.1194/jlr.M054288 PMC4274075

[20] Heikelä M., Vattulainen I., and Hyvönen M. T. (2006) Atomistic simulation studies of cholesteryl oleates: model for the core of lipoprotein particles. Biophys. J. 90, 2247–2257 16399839 10.1529/biophysj.105.069849 PMC1403197

[21] Hall A., Repakova J., and Vattulainen I. (2008) Modeling of the triglyceride-rich core in lipoprotein particles. J. Phys. Chem. B 112, 13772–13782 18844397 10.1021/jp803950w

[22] Cournia Z., Smith J. C., and Ullmann G. M. (2005) A molecular mechanics force field for biologically important sterols. J. Comput. Chem. 26, 1383–1399 16028234 10.1002/jcc.20277

[23] Feller S. E., and MacKerell A. D. (2000) An improved empirical potential energy function for molecular simulations of phospholipids. J. Phys. Chem. B 104, 7510–7515

[24] Schlenkrich M., Brickmann J., MacKerell A. D. Jr., and Karplus M. (1996) An empirical potential energy function for phospholipids: criteria for parameter optimization and applications, in Biological Membranes: A Molecular Perspective from Computation and Experiment (Merz K. M., and Roux B., eds), pp. 31–81, Birkhäuser Boston, Boston, MA

[25] Feller S. E., Yin D., Pastor R. W., and MacKerell A. D. Jr. (1997) Molecular dynamics simulation of unsaturated lipid bilayers at low hydration: parameterization and comparison with diffraction studies. Biophys. J. 73, 2269–2279 9370424 10.1016/S0006-3495(97)78259-6 PMC1181132

[26] Armen R. S., Uitto O. D., and Feller S. E. (1998) Phospholipid component volumes: determination and application to bilayer structure calculations. Biophys. J. 75, 734–744 9675175 10.1016/S0006-3495(98)77563-0 PMC1299748

[27] Kale L., Skeel R., Bhandarkar M., Brunner R., Gursoy A., Krawetz N., Phillips J., Shinozaki A., Varadarajan K., and Schulten K. (1999) NAMD2: greater scalability for parallel molecular dynamics. J. Comput. Phys. 151, 283–312

[28] Grest G. S., and Kremer K. (1986) Molecular dynamics simulation for polymers in the presence of a heat bath. Phys. Rev. A 33, 3628–3631 10.1103/physreva.33.3628 9897103

[29] Feller S. E., Zhang Y., Pastor R. W., and Brooks B. R. (1995) Constant pressure molecular dynamics simulation: the Langevin piston method. J. Chem. Phys. 103, 4613–4621

[30] Darden T., York D., and Pedersen L. (1993) Particle mesh Ewald: an N·log(N) method for Ewald sums in large systems. J. Chem. Phys. 98, 10089–10092

[31] Humphrey W., Dalke A., and Schulten K. (1996) VMD: visual molecular dynamics. J. Mol. Graph. 14, 33–38, 27–28 8744570 10.1016/0263-7855(96)00018-5

[32] Schmidtke P., Le Guilloux V., Maupetit J., and Tufféry P. (2010) fpocket: online tools for protein ensemble pocket detection and tracking. Nucleic Acids Res. 38, W582–W589 20478829 10.1093/nar/gkq383 PMC2896101

[33] Berka K., Hanák O., Sehnal D., Banás P., Navrátilová V., Jaiswal D., Ionescu C. M., Svobodová Vareková R., Koca J., and Otyepka M. (2012) MOLEonline 2.0: interactive web-based analysis of biomacromolecular channels. Nucleic Acids Res. 40, W222–W227 22553366 10.1093/nar/gks363 PMC3394309

[34] Hannuksela M., Marcel Y. L., Kesäniemi Y. A., and Savolainen M. J. (1992) Reduction in the concentration and activity of plasma cholesteryl ester transfer protein by alcohol. J. Lipid Res. 33, 737–744 1619365

[35] Lassel T. S., Guérin M., Auboiron S., Chapman M. J., and Guy-Grand B. (1998) Preferential cholesteryl ester acceptors among triglyceride-rich lipoproteins during alimentary lipemia in normolipidemic subjects. Arterioscler. Thromb. Vasc. Biol. 18, 65–74 9445258 10.1161/01.atv.18.1.65

[36] Kyte J., and Doolittle R. F. (1982) A simple method for displaying the hydropathic character of a protein. J. Mol. Biol. 157, 105–132 7108955 10.1016/0022-2836(82)90515-0

[37] Nguyen H., Pérez A., Bermeo S., and Simmerling C. (2015) Refinement of generalized Born implicit solvation parameters for nucleic acids and their complexes with proteins. J. Chem. Theory Comput. 11, 3714–3728 26574454 10.1021/acs.jctc.5b00271 PMC4805114

[38] Kleinjung J., and Fraternali F. (2014) Design and application of implicit solvent models in biomolecular simulations. Curr. Opin. Struct. Biol. 25, 126–134 24841242 10.1016/j.sbi.2014.04.003 PMC4045398

[39] Zhang L., Sodt A. J., Venable R. M., Pastor R. W., and Buck M. (2013) Prediction, refinement, and persistency of transmembrane helix dimers in lipid bilayers using implicit and explicit solvent/lipid representations: microsecond molecular dynamics simulations of ErbB1/B2 and EphA1. Proteins 81, 365–376 23042146 10.1002/prot.24192 PMC3557542

[40] Hevonoja T., Pentikäinen M. O., Hyvönen M. T., Kovanen P. T., and Ala-Korpela M. (2000) Structure of low density lipoprotein (LDL) particles: basis for understanding molecular changes in modified LDL. Biochim. Biophys. Acta 1488, 189–210 11082530 10.1016/s1388-1981(00)00123-2

[41] Lund-Katz S., Liu L., Thuahnai S. T., and Phillips M. C. (2003) High density lipoprotein structure. Front. Biosci. 8, d1044–d1054 12700101 10.2741/1077

[42] Weinberg R. B., Ibdah J. A., and Phillips M. C. (1992) Adsorption of apolipoprotein A-IV to phospholipid monolayers spread at the air/water interface. A model for its labile binding to high density lipoproteins. J. Biol. Chem. 267, 8977–8983 1577735

[43] Ibdah J. A., Lund-Katz S., and Phillips M. C. (1989) Molecular packing of high-density and low-density lipoprotein surface lipids and apolipoprotein A-I binding. Biochemistry 28, 1126–1133 2496753 10.1021/bi00429a029

[44] Slotte J. P., and Grönberg L. (1990) Oxidation of cholesterol in low density and high density lipoproteins by cholesterol oxidase. J. Lipid Res. 31, 2235–2242 2090717

[45] Zhang L., Song J., Cavigiolio G., Ishida B. Y., Zhang S., Kane J. P., Weisgraber K. H., Oda M. N., Rye K. A., Pownall H. J., and Ren G. (2011) Morphology and structure of lipoproteins revealed by an optimized negative-staining protocol of electron microscopy. J. Lipid Res. 52, 175–184 20978167 10.1194/jlr.D010959 PMC2999936

[46] Ren G., Rudenko G., Ludtke S. J., Deisenhofer J., Chiu W., and Pownall H. J. (2010) Model of human low-density lipoprotein and bound receptor based on cryoEM. Proc. Natl. Acad. Sci. U.S.A. 107, 1059–1064 20080547 10.1073/pnas.0908004107 PMC2798884

[47] Elder R. M., and Jayaraman A. (2012) Sequence-specific recognition of cancer drug-DNA adducts by HMGB1a repair protein. Biophys. J. 102, 2331–2338 22677386 10.1016/j.bpj.2012.04.013 PMC3353062

[48] Wang S., Wang X., Deng L., Rassart E., Milne R. W., and Tall A. R. (1993) Point mutagenesis of carboxyl-terminal amino acids of cholesteryl ester transfer protein. Opposite faces of an amphipathic helix important for cholesteryl ester transfer or for binding neutralizing antibody. J. Biol. Chem. 268, 1955–1959 7678413

[49] García-González V., Gutiérrez-Quintanar N., Mendoza-Espinosa P., Brocos P., Piñeiro A., and Mas-Oliva J. (2014) Key structural arrangements at the C-terminus domain of CETP suggest a potential mechanism for lipid-transfer activity. J. Struct. Biol. 186, 19–27 24530617 10.1016/j.jsb.2014.02.002

[50] McLean L. R., and Phillips M. C. (1984) Kinetics of phosphatidylcholine and lysophosphatidylcholine exchange between unilamellar vesicles. Biochemistry 23, 4624–4630 6498159 10.1021/bi00315a017

[51] Lauer M. E., Graff-Meyer A., Rufer A. C., Maugeais C., von der Mark E., Matile H., D'Arcy B., Magg C., Ringler P., Müller S. A., Scherer S., Dernick G., Thoma R., Hennig M., Niesor E. J., et al (2016) Cholesteryl ester transfer between lipoproteins does not require a ternary tunnel complex with CETP. J. Struct. Biol. 194, 191–198 26876146 10.1016/j.jsb.2016.02.016

