# Supplementary material for: Insights into the Tunnel Mechanism of Cholesteryl Ester Transfer Protein through All-atom Molecular Dynamics Simulations
Source: J Biol Chem. 2016 May 3;291(27):14034–44. doi: 10.1074/jbc.M116.715565 (PMC4933163; doi:10.1074/jbc.M116.715565)
Supplement: Supplemental Data [file supp_291_27_14034__index.html]

Insights into the tunnel mechanism of cholesteryl ester transfer protein using all-atom molecular dynamics simulations — Insights into the Tunnel Mechanism of Cholesteryl Ester Transfer Protein through All-atom Molecular Dynamics Simulations — CETP Tunnel Mechanism by MD Simulations — Supplemental Data 

# Insights into the Tunnel Mechanism of Cholesteryl Ester Transfer Protein through All-atom Molecular Dynamics Simulations

## Supplemental Data

- Supporting video 1 (.avi, 22.9 MB) - All-atom MD simulation of CE transferring through a CETP molecule under a representative driving force of 11 kcal/mol/?. The POPC head groups and fatty tails are colored yellow and green, respectively, whereas the CE, TG, CETP and water molecules are colored pink, gray, blue and red, respectively.
